# Work motivation and factors associated with it among health professionals in Debre Markos Comprehensive Specialized Hospital

**DOI:** 10.1038/s41598-024-52409-5

**Published:** 2024-01-29

**Authors:** Eniyew Tegegne, Yikeber Argachew Deml, Getasew Yirdaw, Yenewa Bewket

**Affiliations:** 1 Department of Environmental Health, College of Medicine and Health Science, Debre Markos University, Debre Markos, Ethiopia; 2 Department of Biomedical Sciences, College of Medicine and Health Science, Debre Markos University, Debre Markos, Ethiopia

**Keywords:** Health policy, Health services, Occupational health, Public health

## Abstract

Motivation is the level of a person's willingness to put forth and maintain an effort in support of organizational goals. However, motivation towards task execution is affected by the organization and individual goals. For instance, low morale among the staff can damage the quality of service delivery. Hence, this study was intended to assess the working motivation status and factors associated with it among health professionals at Debre Markos Comprehensive Specialized Hospital. A hospital-based cross-sectional study was employed. Stratified sampling techniques were used to extract sample from each job category proportionally. To make the distribution fair, all health workers were grouped according to their job title and selected by using the lottery method from each group. A standardized, self-administered questionnaire was used to collect data. Data was checked, coded, and entered into EpiData 3.1 and exported for analysis into SPSS 25. Variable in the multivariable logistic regression model with a p value of < 0.05 at 95% CI were taken as significantly associated to motivation status. A total of 319 people were involved, with a 100% response rate. 20.4% of health professionals were motivated at Debre Markos Comprehensive Specialized Hospital. Job satisfaction (AOR 6.46, 95% CI 1.72, 24.35), the presence of adequate medical supplies (AOR 5.01, 95% CI 1.23, 25.37), work place security (AOR 6.78, 95% CI 1.498, 30.72), and the presence of training opportunities in health facilities (AOR 2.23, 95% CI 1.01, 4.96) were significant factors associated with motivation status. The proportion of motivated health professionals was very low compared to previous studies in Ethiopia. The presence of security at work, adequate medical equipment, drugs, and supplies, job satisfaction, and the presence of training opportunities were predominant motivational factors. The hospital administration needs to give priority and work to safeguard security, ensure adequate medical supplies, and offer training to improve their satisfaction and motivation.

## Introduction

A conducive work environment is indispensable to the health and safety of workers and their efforts to achieve organizational missions. Work motivation is the level of a person's willingness to put forth and maintain an effort in support of organizational goals. Human resources are one of the most decisive determinants of quality health care delivery^1^. Yet, the world is facing a shortage of about 4.2 million healthcare workers^2^. According to the Global Health Workforce Alliance (GHWA), many countries fall below the threshold of 2.3 skilled health workers per 1000 population, particularly sub-Saharan Africa, including Ethiopia, where the density of skilled professionals per 1000 population is 0.3^3^, where their motivation is paramount for effective and efficient utilization of available workforces.

Out of the world's disease burden, sub-Saharan Africa bears the largest share, where local health systems are ineffective, unfair, and even treacherous. Health professionals' motivation has been identified as the primary determinant of the quality of health-care services, despite the fact that there are other causes for this underperformance^4^. Governments place a lot of attention on developing health infrastructure and enhancing the supply chain for medical supplies, but these efforts will be ineffective if the health workforce is not motivated^5^.

Ethiopia has been making significant reforms to the whole health sector, including business treating and re-engineering, health care finance, and health information technology^6^. Despite the sector's human resource crisis, 73 percent of doctors and 82 percent of nurses work in the public sector^7^. However, the public health sector, which makes heavy use of human resources, is unsuccessful and disorganized, and the management of human resources in this sector has had a negative impact on the delivery of health services^8^.

Due to a variety of factors, Ethiopia's efforts to achieve the desired outcomes were not as successful as expected. The one is a shortage of human resources in the health system. Since motivated health workers are more likely to work for profit in private and non-governmental organizations, many trained professionals are moving abroad or leaving the public sectors^9^. For instance, work place violence leads to a high rate of turnover intention^10^ and decreased job satisfaction^11^ which would affect workers motivation.

Even though it is planned to train more doctors to fill a gap of over 80% in Ethiopia, said Ethiopia’s assistant health ministers at an international conference in Uganda to tackle the global shortage in the health workforce, doctors still have been leaving the country to earn better salaries offered in the United States and some rich African countries, such as Botswana^12^. Despite efforts to address this gap, there has been a wave of protests among medical students in various university hospitals in Ethiopia, which are a showcase for unmet needs.

Studies on health worker motivation and determinant factors that affect their motivation were understudied in developing nations as well as in Ethiopia. The motivation score of health workers in West Amhara public hospitals was 58.6%^13^, and 48.6% of HEWs^14^ were satisfied with their job in Ethiopia. These characteristics have a direct impact on the quality of healthcare provided. The subject of how to increase the motivation of health workers has generated interest, but little attention has been paid to it. Fewer studies have been done concentrating on the job satisfaction of health workers in hospital settings rather than factors affecting motivation status in Ethiopia. To clearly put the research question, "Why are health workers not motivated to work?" Hence, the purpose of this study was to determine the work motivation status and its associated factors among health professionals at Debre Markos Comprehensive Specialized Hospital (DMCSH), which will be applied to health care settings in the same context.

## Materials and methods

### Study design and settings

A hospital-based cross-sectional study was conducted at Debre Markos Comprehensive Specialized Hospital, which is located in Amhara regional state in Debre Markos town, the capital of the East Gojjam zone, 299 km northeast of Addis Ababa and 265 km from Bahir Dar.

The hospital was established in 1957 E.C., and it serves about 5 million people coming from East Gojjam, West Gojjam, Awi, and parts of the Oromia region. The hospital provides different services, including outpatient and inpatient treatment and care services for the community. It has a total of 221 beds in the major and minor departments: surgery (36 beds), internal medicine (44 beds), obstetrics and gynecology (40 beds), pediatrics (4 beds at ETAT for kept patients), 44 beds at the ward, neonatal Intensive Care Unit (ICU) (20 beds), and adult ICU (4 beds) for inpatient admission services. In minor wards, the ophthalmology unit has 18 beds, and psychiatry has 11 beds for patient management.

The hospital has a total of 489 health workers, among them 58 physicians, 2 dentists, 17 Masters of Science (MSc), 2 Integrated Emergency Surgical Officers (IESO), 43 pharmacists (15 Bachelor of science (BSc), and 28 diplomas), 10 Health Officer (HO), 8 (Health Information Technologist (HIT) (1 BSc and 7 diplomas), 44 midwives (32 BSc and 12 diplomas), 230 nurses (206 BSc and 24 diplomas), 2 environmental health, 9 anesthesia professionals (6 BSc and 3 diplomas), 32 laboratory professionals (21 BSc and 11 diplomas), 10 radiology (4 BSc and 6 diplomas), 2 physiotherapy, 6 psychiatry nurses, 5 optometry professionals, 3 ophthalmic professionals, 1 cataract, 4 Emergency Medical Technicians (EMT), 1 biomedical diploma The average number of patients admitted to the hospital per month is 1493, according to the Debre Markos Comprehensive Specialized Hospital (DMCSH) admission office report for May 2021. The study was conducted from May 2021 to July 2021.

### Source population and study population

The source population were health professionals working at DMCSH.

### Inclusion and exclusion criteria

#### Inclusion criteria

All health professional who were working in DMCSH were included.

#### Exclusion criteria

Those who were on annual leave and those, and recently recruited (less than six months) during the time of data collection were excluded.

### Sample size and sampling techniques

#### Sample size determination

The sample size was computed by using single population proportion formula by taking the following assumptions in to consideration:

P = 25.1% taken from a research conducted in Jimma Medical Menter^2^,

5% margin of error (d).

Non-response rate of 10%

95% confidence level (Z = 1.96)

$${\text{n}} = \left( {{\text{Za}}/2} \right)^{2} {\text{P}}(1 - {\text{P}})/{\text{d}}^{2}$$

$${\text{n}} = \left( {{1}.{96}} \right){2 }0.{251}\left( {{1} - 0.{251}} \right)/\left( {0.0{5}} \right){2}$$

n = 290.

Then, 10% of the non-response rate which was 290(0.1) = 29 and the final sample size was 290 + 29 = 319.

#### Sampling technique

**Figure 1:**
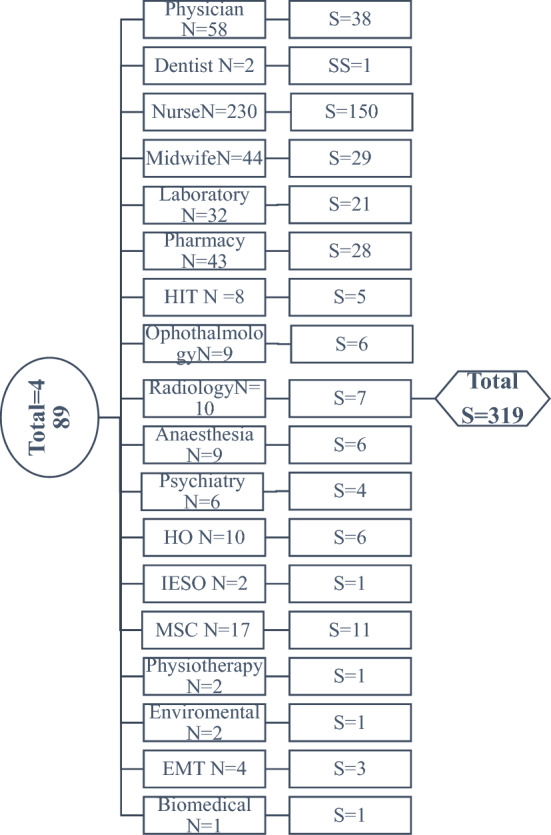
Sampling procedure from health workers at Debre Markos Comprehensive Specialized Hospital, 2021.

Stratified sampling technique was used from the total of 489 HWs and strata formed by their professions as physician, Nurse, Laboratory, Pharmacy, Midwifery, Radiology, Ophthalmology, HO and Health Informatics. After stratification, proportional allocation was done based on the number of workers in each strata. Subsequently, actual study participants from each stratum were selected using the lottery method (Fig. 1).

### Operational definitions

*Motivation:* The extent to which a person feels good about themselves when they do their jobs well.

*Intrinsic motivation:* Work motivation for healthcare professionals in health centers is characterized by intrinsic motivational factors, such as job pleasure, recognition for accomplishment, advancement, and others.

*Extrinsic motivation:* Extrinsic motivational variables, such as management, supervision, the workplace environment, and others, produce the job motivation of healthcare workers.

*Job satisfaction:* The level of favorability with which the healthcare professionals see their work in this study.

*Recognition:* Indicates medical professionals who feel that they are making a difference at the medical facility. Monitoring the performance of healthcare professionals both horizontally and vertically within an organization^15^.

*Advancement:* Is the acquisition of new knowledge and abilities; improved job satisfaction (more challenging work, greater variety etc.) connecting work with personal goals and aspirations; giving employees a road map for possible lateral or vertical career advancement.

*Achievement:* A work must give healthcare workers the chance to showcase their range of skills and should be enriching enough to improve motivation and performance.

### Variables

Dependent (outcome) variable: Motivation status.

Independent (exposure) variables: The independent variables were composed of eight sections: sociodemographic, achievement (individual success), job satisfaction, advancement, recognition, management, supervision, and work environment factors. All variables were designed to be categorical, including continuous variables like age, salary, and service year.

#### Data collection tool and procedure

Data were gathered using a self-administered, well-structured questionnaire. It was given to be filled out by the study participants themselves (self-reported). The questionnaire was translated into the local language and then back into English to ensure consistency. The questionnaire was developed for measuring general motivation and satisfaction from a prior study as well as from a document entitled a Guide to Improving Organizational Performance^16^, and was validated (Cronbach's alpha = 0.84).

Multi-dimensional work motivation scales were used to evaluate employee motivation (dependent variable) for the job. Individual success, job satisfaction, advancement, recognition, management, supervision, and work environment were the domains that were divided into the work motivation questionnaire's components. The motivation subsidiary scores for each area ranged from strongly disagree (1) to strongly agree (5), measured using the Likert scale of (5). The score might have been anywhere between 1 and 5. By averaging the results of each subscale, the overall motivation score was calculated. The mean (average) value across all domains was derived to assess each person's level of work motivation. To ascertain if a healthcare practitioner was driven to perform his or her duties or not, the mean value of the domains was used as a cut-point value based on previous studies so that comparison of the study finding would be possible. As a result, healthcare professionals with scores below the mean were viewed as unmotivated, while those with scores above the mean were seen as motivated^17^.

### Data quality control

The training for data collectors was given for one day regarding the purpose of the study, the importance of participant privacy and data integrity, techniques for reducing bias, or strategies for handling potential participant questions or concerns. The lead investigator reviewed questionnaires to look for errors and missing information. After that, responses were carefully coded and verified. The supervisors had given the completed questionnaire to the lead investigator after ensuring that every questionnaire was consistent and comprehensive. Additionally, 5% of the questionnaire was pretested at Debre Markos town's four health centers before the actual study began to ensure its accuracy, simplicity, and soundness. Based on the pretest findings, some categorical variables had been made to be mutually exclusive and exhaustive. Its face validity, or whether the test's content seems appropriate for the goals that were thought to be included in it, and its content validity, or whether the test is completely representative of what it seeks to measure, were ensured. The reliability was checked by Cronbach’s alpha (P value = 0.88).

### Data analysis procedure

After the data is collected, it will be reviewed for accuracy and consistency, coded, and entered into Epi Data 3.1. For the analysis, the data was exported to SPSS 21. Model fitness was assessed using the Hosmer and Lemeshow goodness of fit tests (p-value = 0.8)^18^. Bivariate (*Y* = *α* + *bX* + *ε*)^19^ and multi-variable binary logistic regression model (*Y* = *a* + *b*_*1*_*X*_*1*_ + *b*_*2*_*X*_*2*_ + ⋯ + *b*_*n*_*X*_*n*_)^19^ were run to analyze the responses to each question and determine the significance of the relationship between the dependent and independent variables. For the multivariate analysis, independent variables with a P-value of less than 0.25 in the bivariate regression analysis were a possibility. A p value of < 0.05 was used to determine statistical significance. Finally, the results of the study were presented using narration, tables, and figures.

### Ethical approval

All the methods were carried out in accordance with relevant guidelines and regulations. Ethical approval for the study was obtained from the Ethical Review Committee of Debre-Markos University, College of Medicine and Health Sciences, with an ethical approval number of HSC/R/C/Ser/PG/Co/132/12/14. Permission was obtained from the Debre Markos Comprehensive Specialized Hospital, and written informed consent was obtained from each study participant. Health workers were informed of the purpose of the study, and the right to refusal and withdrawal from the study was respected.

## Results

### Sociodemographic characteristics of study participants

A total of 319 healthcare workers were involved in this study. They correctly filled out and returned the questionnaire, giving a response rate of 100%. Of the total study participants, 185 (58%) were male. The mean age of respondents was 34.78 (SD ± 6.43) years. About one-third of respondents, 108 (58.9%), were in the age range of 24 to 29 years. The median service year of respondents was 3 years. About half of the study participants, 158 (49.5%), were married. More than half (186, or 58.3%) of respondents' monthly salaries ranged between 4610 and 8017 Ethiopian birr, followed by 96 (30.1%), which ranged from 8018 to 11,305 Ethiopian birr. About half of the respondents, 149 (46.7%), were nurses by profession. Two-thirds of the respondents, 223 (69.9%), were first-degree holders (Table 1).

**Table 1:** Socio-demographic characteristics and binary logistic of health professionals at Debre Markos Comprehensive Specialized Hospital, July 2021.

Variables	Categories	Frequency (n)	Percent
Gender	Male	185	58.5
	Female	134	42.0
Age	< 24	8	2.5
	24–29	108	33.9
	30–35	139	43.6
	36–40	43	13.5
	> 41	21	6.6
Profession	Doctor	37	11.6
	Nurse	149	46.7
	Laboratory	21	6.6
	Health officer	6	1.9
	Midwife	33	10.3
	Pharmacy	28	8.8
	Others	45	14.1
Monthly salary	1100–4609	20	6.3
	4610–8017	186	58.3
	8018–11,305	96	30.1
	> 11,305	17	5.3
Marital status	Married	158	49.5
	Unmarried	138	43.3
Educational level	MSc	53	16.6
	Degree	223	69.9
	Diploma	31	9.7
Service years	< 2	16	5.0
	2–5	120	37.6
	6–10	133	41.7
	> 10	50	15.7

### Health professionals’ motivation status

**Figure 2:**
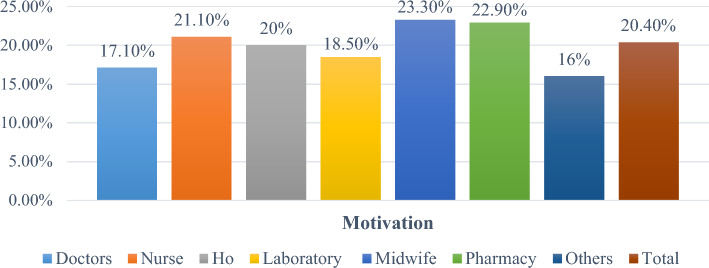
Debre Markos Comprehensive Specialized Hospital health professions motivational status by professions category, July 2021.

The percentage of health professionals motivated was 20.4% (CI: 15.98%, 24.82%). This motivation level varied between profession categories, from least 16% (anesthetists, radiographers, and psychiatry) to the highest 23.3% in midwifery, respectively (Fig. 2).

### Variables of the study

Frequency, mean, standard deviation, minimum and maximum values were computed for variables to provide a summary of the data. Job happiness, management, work environment, recognition, achievement, advancement, and motivational variables all have their own items to help with interpretation clarity. The scale for negatively worded questions was reverse coded so that 1 was ‘strongly agree’ and 5 ‘strongly disagree’. Thus, a high score shows disagreement with a negative statement and is therefore suggestive of higher motivation (Table 2).

**Table 2:** Summary statistics for variables on health professionals of Debre Markos Comprehensive Specialized Hospital, July 2021 (N = 319).

Variables	Mean score	SD
Motivation	2.8077	0.95530
Satisfaction	2.9922	0.65744
Supervision	2.8966	0.8589
Recognition	3.0090	0.50845
Achievement	2.9189	0.51485
Advancement	2.6934	0.91042
Management	3.0474	0.68081
Environment	2.9881	0.60082

### Factors associated with motivation

The final model of binary logistic regression showed that security, equipment, drugs, and supplies within the hospital, job satisfaction, and training were found to be independently associated with working motivation among health care workers in DMCSH. The odds of being motivated were about six (AOR 6.46, 95% CI 1.71, 24.34) times higher among health professionals who were satisfied by their job than their counterparts who were not satisfied. The odds of being motivated were four (AOR 4.006, 95% CI 1.23, 25.37) times higher among those health professionals where equipment, drugs, and supplies were timely supplied, and workers who believe that there is security were about six (AOR 6.78, 95% CI 1.45, 30.72) times more likely to be motivated than those who believe they are not secured at work. Workers who were offered regular training in health facilities were two (AOR 2.23, 95% CI 1.01, 4.96) times more likely to be motivated than their untrained counterparts (Table 3).

**Table 3:** Bivariate and multivariate logistic regression of DMCSH health professional’s motivation and its associated factors, June 2020.

Variables	Categories	Percentage (%)	COR (95% CI)	P-value	AOR (95% CI)
Job satisfaction	Yes	45.1	10.67 (5.47,20.79)	0.01	6.46 (1.72,24.35)
	No	54.9	*Ref*		*Ref*
Presence of training opportunities	Ye	30.7	1.74 (1.1, 2.9)	0.05	2.23 (1.01, 4.96)
	No	69.3	*Ref*		*Ref*
Heavy workload	Yes	85.9	1.58 (1, 0.54)	0.42	1.37 (0.63, 2.99)
	No	14.1			
Salary	Adequate	21.9	1.68 (1, 2.8)	0.33	0.66 (0.28, 1.53)
	Not adequate	78.1	*Ref*		*Ref*
Advancement	Advanced	41.7	0.5 (0.30, 0.82)	0.98	0.99 (0.46, 2.15)
	Non advanced	58.3	*Ref*		*Ref*
Management style	Democratic	61.4	1.5 (0.9, 2.4)	0.22	1.59 (0.76, 3.33)
	Non democratic	38.6	*Ref*		*Ref*
Justice and fairness	Fair	38.2	0.25(0.14,0.44)	0.5	0.69(0.23,2.03)
	Unfair	61.8	*Ref*		*Ref*
Work place	Appropriate	63.0	0.26 (0.14,0.5)	0.96	1.03 (0.28,3.77)
	Not appropriate	37.0	*Ref*		*Ref*
Medical equipment, drugs and supplies	Adequate	36.7	24.98 (7.85,75.39)	0.00	4.01 (1.23,25.37)
	Not adequate	63.3	*Ref*		*Ref*
Interpersonal relationship	Good	63.6	0.066 (0.020,0.217)	0.32	2.79 (0.37,21.38)
	Not good	36.4	*Ref*		*Ref*
Secured at work place	Yes	47.3	89.44 (30.68,260.75)	0.02	6.78 (1.5,30.72)
	No	52.7	*Ref*		*Ref*

*Ref reference.*

## Discussion

Health workers' motivation has been pointed out as a highly important factor for effective health service delivery. This study showed that the general motivation level of the DMCSH health profession was 20.4% (95% CI 15.98%, 24.82%). The motivation status in the current study is comparable to the study done at Jimma Medical Center, which revealed a 25.1% overall motivation level among health professionals, even though it is slightly lower. The time difference when the research was done and the economic inflation during the period of this study might be mentioned as reasons for the observed discrepancy. The cost of goods is rising for many production and service-giving industries in the country after ethnic-based violence, displacement, and chaos prevailing in the country. Although health professionals will be devastated by brutal ethnic-based conflicts that are overburdening health care settings and causing violence against health care in conflict-prone areas^20^. On the other hand, the similarity might be due to nearly similar income and allowance, geographical similarity, similarity in methodology, a nearly similar sample size, and the sampling method used^21^. Also, it is lower than the study carried out in public hospitals in West Amhara, which revealed a proportion of 58.6%^13^, and 55.5% of nurses in Jimma University^22^. The difference may be brought on by variations in the study's duration, location, methodology, sample size, and sampling technique, and study population^16^.

The current study shows that adequate medical equipment, drugs, and supplies were one of the factors found to affect health professionals’ motivation. This finding was consistent with the study done at Jimma Medical Center^22^. This in turn implies that the availability of medical supplies needs to be ensured in order to satisfy their clients' requirements so that appropriate prescriptions and diagnostics, as well as other promotion interventions, can be offered for a better outcome^23^.

The presence of security at work was found to be the main motivating factor in this study. This finding is consistent with studies done at Addis Ababa University, public hospitals in West Amhara, Jimma University, and Indian states^13,22,24,25^. Workplace security is put in place to keep people, property, and information safe from both physical and virtual dangers^26^. Work place violence impedes workers job satisfaction, and hence will affect their motivation to work^27–29^. These dangers can take many forms, from digital security hazards like cyberattacks, data breaches, and hacking to theft, violence, and sabotage, which will ultimately affect health service performance^30^.

The odds of job satisfaction were also higher among motivated than tamong non-motivated staffs. This finding is further cemented by the prior studies conducted in Jimma University^2^, and Pakistan^30^. Job satisfaction is a complex phenomenon with many different meanings and viewpoints^31^. Despite research showing a connection and dependence between motivation, job happiness, and performance, the relationship is circular: Motivation causes job happiness and performance; happiness and performance cause motivation^32,33^. Poor salaries would be the flaming barrier to the poor satisfaction of health professionals, which will induce them to migrate to Ethiopia looking for better salaries^12^. This implies that people may not know the true value of their health until it is impaired, upon which the government of Ethiopia should work.

Regularly, the presence of training in health facilities is also a significant predictor that is found to be associated with the motivation of health professionals. This is similar to a study conducted at Addis Ababa University^25^, Jimma University^22^, and Indonesia^34^. The kind of non-financial incentives identified should be taken into consideration when developing measures to improve the motivation of workers. Training and development programs help to enhance employee performance^35^, enhance employee yield, lessen worker turnover^36^, and advance company culture. Continuous professional development through training is nonstop and never-ending in nature, so it shall be given to health professionals^37^.

The study is cross-sectional in design, where the temporal relationship between dependent and independent variables is impossible, and solely employs quantitative analysis, which makes it difficult to understand the context of the phenomenon.

## Conclusion and recommendation

The findings of this study showed that health professions' motivation status was very low in both the overall and sub professional categories. The presence of adequate medical equipment, drugs, and supplies, security at work, training, and job satisfaction were significant predictors of motivation status. Health authorities need to ensure the availability and quality of hospital medical equipment and supplies and thrive to fulfill security issues, offer them with continuous professional development to workers to create a conducive and enabling work environment for a better mutual outcome.

### Supplementary Information

Supplementary Information.

## Data Availability

The dataset is accessible at the corresponding author upon reasonable request.

## Abbreviations

AOR: Adjusted odds ratio
COR: Crude odds ratio
DMCSH: Debre Markos Comprehensive Specialized Hospital
HO: Health officer
BSc.: Bachelor of science
IESO: Integrated emergency surgical officers
HIT: Health information technologist
EMT: Emergency medical technicians
ICU: Intensive care unit
