# Supplementary material for: Work motivation and factors associated with it among health professionals in Debre Markos Comprehensive Specialized Hospital
Source: Sci Rep. 2024 Jan 29;14:2381. doi: 10.1038/s41598-024-52409-5 (PMC10825199; doi:10.1038/s41598-024-52409-5)
Supplement: Supplementary file 1 — Supplementary Information. [file 41598_2024_52409_MOESM1_ESM.docx]

### **Questionnaire**

Please read all the questions carefully. The survey has been designed to facilitate easy answering. Answer requires either a √ or □ to indicate your appropriate responses. All Answers are confidential. Only the researcher sees and processes the questionnaire.

**Part I: Socio-demographic data**

1. Sex? A. Male B. Female
2. Age? ______________
3. What is your current marital status? A. Married B. Unmarried C. Divorced D. Widows
4. Your educational level (Status)? A. Msc B. Degree C. Diploma D. Others specify
5. Your service years? ____________
6. What is your job title? A. Doctor B. Nurse C. HO D. Laboratory E. Midwife F. Pharmacist G. Others specify
7. Your monthly salary? ___________

**Part II: Factors affecting motivations**

Indicate your responses to the following statements regarding individual motivational assessment.

8. Please read each item in the following statements, and then indicate with an X in the appropriate answer box, according to the following code definitions:

1. Strongly disagree

2. Disagree

3. Uncertain

4. Agree

5. Strongly agree

| **Statements** | 1 | 2 | 3 | 4 | 5 |
| --- | --- | --- | --- | --- | --- |
| You feel motivated to work hard |  |  |  |  |  |
| Only do this job to get paid |  |  |  |  |  |
| My job is a means to be secured for a long time |  |  |  |  |  |
| I am very satisfied with my job |  |  |  |  |  |
| I feel my work contributes to the organization’s performance |  |  |  |  |  |
| The work I do gives me a feeling of personal achievement |  |  |  |  |  |

1. Please read each item in the following statements, and then indicate with an X in the appropriate answer box, according to the following code definitions:

1. Strongly disagree

2. Disagree

3. Uncertain

4. Agree

5. Strongly agree

| **Statements** | 1 | 2 | 3 | 4 | 5 |
| --- | --- | --- | --- | --- | --- |
| You are satisfied with your remuneration |  |  |  |  |  |
| My job provides me with opportunities for advancement to higher levels jobs. |  |  |  |  |  |
| I am not satisfied with my colleagues in my work |  |  |  |  |  |
| I am satisfied with my supervisor |  |  |  |  |  |
| I am satisfied with the opportunity to use my abilities in this job |  |  |  |  |  |
| I am satisfied that I accomplish something worthwhile in this job |  |  |  |  |  |
| I am a hard worker |  |  |  |  |  |
| Do things that need doing without being asked or told |  |  |  |  |  |
| My colleagues value my contribution. |  |  |  |  |  |
| My manager/supervisor gives me regular, timely feedback that  helps me improve my performance |  |  |  |  |  |
| This organization provides me with skills and knowledge that will benefit my future career |  |  |  |  |  |

**10.** Please read each item in the following statements, and then indicate with an X in the appropriate answer box, according to the following code definitions:

1. Strongly disagree

2. Disagree

3. Uncertain

4. Agree

5. Strongly agree

| **Statements** | 1 | 2 | 3 | 4 | 5 |
| --- | --- | --- | --- | --- | --- |
| I do not think that my work in this health facility is valuable these days |  |  |  |  |  |
| I am proud to be working for this health facility |  |  |  |  |  |
| I find that my values and this health facility are very similar |  |  |  |  |  |
| I am glad that I work for this facility rather than other facilities in the country |  |  |  |  |  |
| I feel very little commitment to this health facility |  |  |  |  |  |
| This health facility really inspires me to do my very best on the job |  |  |  |  |  |
| I am punctual about coming to work |  |  |  |  |  |
| I am often absent from work |  |  |  |  |  |
| It is not a problem if I sometimes come late for work |  |  |  |  |  |

Indicate your responses to the following statements regarding to organizational level motivational assessment.

11. Please read each item in the following statements, and then indicate with an X in the appropriate answer box, according to the following code definitions:

1. Strongly disagree

2. Disagree

3. Uncertain

4. Agree

5. Strongly agree

| **Statements** | 1 | 2 | 3 | 4 | 5 |
| --- | --- | --- | --- | --- | --- |
| My job duties, requirements, and goals are clear and specific. |  |  |  |  |  |
| The health center promotion criteria are clear and fair |  |  |  |  |  |
| The relationship between management and staff of this health  Centre cordial |  |  |  |  |  |
| My managers/supervisor inspires me to do my best. |  |  |  |  |  |
| Work with skilled competent people who are good at their jobs. |  |  |  |  |  |
| People in this organization have a shared sense of purpose. |  |  |  |  |  |
| I do not like the way the organization operates |  |  |  |  |  |
| The way things are organized around here makes it hard for people to do their best work. |  |  |  |  |  |
| I am delighted to tell people that I work for this organization |  |  |  |  |  |

12. Please read each item in the following statements, and then indicate with an X in the appropriate answer box, according to the following code definitions:

1. Strongly disagree

2. Disagree

3. Uncertain

4. Agree

5. Strongly agree

| **Statements** | 1 | 2 | 3 | 4 | 5 |
| --- | --- | --- | --- | --- | --- |
| This organization’s mission is understood by everyone who works here. |  |  |  |  |  |
| The people I work with are comfortable in suggesting changes and improvements to each other. |  |  |  |  |  |
| I am clear about the objectives I need to achieve. |  |  |  |  |  |
| I trust and respect my immediate supervisor. |  |  |  |  |  |
| My manager emphasizes my positive contributions when reviewing my performance. |  |  |  |  |  |
| There is a great deal of cooperation between people in this organization. |  |  |  |  |  |
| I am given enough authority to allow me to do my job effectively. |  |  |  |  |  |
| If I have an idea for improving the way we do our work my supervisor/manager will usually listen to me. |  |  |  |  |  |

13. Please read each item in the following statements, and then indicate with an X in the appropriate answer box, according to the following code definitions:

1. Strongly disagree

2. Disagree

3. Uncertain

4. Agree

5. Strongly agree

| **Statements** | 1 | 2 | 3 | 4 | 5 |
| --- | --- | --- | --- | --- | --- |
| Good opportunities for continuing education are available |  |  |  |  |  |
| The necessary training is given to ensure job effectiveness. |  |  |  |  |  |
| Job specific refresher courses are available. |  |  |  |  |  |
| In-service training adequately addresses the skill gaps. |  |  |  |  |  |
| Incompetent health care providers are identified and provided with the necessary support. |  |  |  |  |  |
| Adequate medical equipment’s, drugs and supplies |  |  |  |  |  |
| Your work performed perception is well done |  |  |  |  |  |
| The organization is fair for all health workers |  |  |  |  |  |

14. Please read each item in the following statements, and then circle your choice:

| Statements | Responses | |
| --- | --- | --- |
| What about the work place? | Appropriate | Not appropriate |
| Medical equipment’s, drugs and supplies | Adequate | Not adequate |
| Interpersonal relationships | Good | Not good |
| Justices and fairness | Fair | Unfair |
| Satisfied to work | Yes | No |
| Secured at work place | Yes | No |
| Advancement | advanced | Not advanced |
| Presence of training opportunities | Yes | No |
| Management style | democratic | Non democratic |
| Heavy work load | Yes | NO |
| Salary | adequate | Not adequate |

**THANK YOU!!**
